# Vaccination of Elderly People Affected by Chronic Diseases: A Challenge for Public Health

**DOI:** 10.3390/vaccines10050641

**Published:** 2022-04-19

**Authors:** Francesco Paolo Bianchi, Silvio Tafuri

**Affiliations:** Interdisciplinary Department of Medicine, Aldo Moro University of Bari, 70121 Bari, Italy; dr.francesco.bianchi@gmail.com

Elderly people have a limited regenerative capacity and are more susceptible to disease, syndromes, injuries, and illnesses than younger adults. There are many causes for this phenomenon, including anatomical and physiological changes and increased risk from hospitalization or invasive procedures, but most importantly, age-related changes in the functionality of the immune system, summarized as immunosenescence, also play a significant role [1]. The immune system equally suffers from the effects of biological aging, exhibiting a progressive decline in function that collectively results in diminished humoral and cellular immune responses [2,3,4,5].

In addition to alterations attributable to immunosenescence, other factors such as chronic disease, obesity, nutrition, frailty, functional status, or stress can affect immune function and further compromise the immune response to vaccination in older adults [6]. The presence of one or two chronic diseases is associated with a 40- to 150-fold increase in the incidence rate of influenza or pneumonia [6]. In addition, it is necessary to specify that other conditions (geriatric syndromes) make the elderly subject at greater risk for infection [6].

Thus, these conditions contribute to increased susceptibility to infectious diseases in the elderly, some of which may be vaccine-preventable [6]. Vaccinations are the primary strategy to prevent viral and bacterial infections (e.g., influenza, respiratory syncytial virus, herpes zoster, pneumococcal disease) that are most frequent among older adults compared with younger individuals [6]. As provided by the Centers for Diseases Control and Prevention, the recommended vaccinations for older adults are the seasonal influenza vaccine, the periodical booster Td or Tdap (tetanus, diphtheria, and pertussis) vaccine, the Herpes zoster vaccine (recommended for healthy adults 50+ years), and the pneumococcal vaccine [7]. Moreover, a specific vaccine prophylaxis protocol is recommended for older adults with chronic conditions, such as asplenia [8], diabetes [9], cardiovascular diseases [10], HIV infection [11], liver diseases [12], lung diseases [13], renal diseases [14], and a weakened immune system [15]. As of December 2020, the anti-COVID19 vaccine is also recommended in this category [16]. Specific recommendations and protocols are provided by the European Centre for Disease Control and Prevention as well [17]. Nevertheless, primary vaccine responses are often lower in this population, frequently fail to induce long-term protective immunity, and place these individuals at further risk for subsequent disease; these findings have been predominantly linked to the function and perceived failure of the adaptive immune response in older adults, as described above [6]. Vaccine prophylaxis in elderly adults at higher risk for infection is a topic under study, and more evidence can be found as knowledge advances. As reported above, specific protocols for these population sub-groups are recommended [8,9,10,11,12,13,14,15,16]. Unfortunately, the evidence reported in the literature shows that the vaccination coverage achieved in these population sub-groups is not satisfactory [18,19,20,21,22].

In Italy, according to the ISTAT archive for the year 2021, almost 14,000,000 inhabitants are 65 years of age and older; no official data are Available online how many of them are affected by a chronic condition and, thus, how many of them are at higher risk of infectious disease complications. Nevertheless, a 2021 study estimated that in Apulia, an Italian Region with 4 million inhabitants, a proportion of citizens with at least one underling condition to be around 65%; this proportion increases with increasing age [23]. A 2022 Italian study [24] reported that, among 852,211 living subjects residing in Veneto Region and aged between 70 and 100 years, the prevalence of subjects with at least one chronic disease ranged from 69% to 74%. Of course, additional studies are needed to estimate these data on whole the Italian population aged 65+ years. So, the 2017–2019 Italian National Preventive Vaccination Plan, following the guidelines described above by the Centers for Disease Control and Prevention [7], recommended that individuals aged ≥65 years receive the anti-influenza vaccines, anti-pneumococcal vaccines (13-valent conjugate followed by 23-valent polysaccharide), anti-Herpes zoster, the booster of anti-tetanus vaccine, anti-meningococcal, hepatitis A, hepatitis B, measles- mumps-rubella (MMR), and Varicella (Vzv) vaccine if special risk conditions are diagnosed [25]. As of December 2020, the anti-COVID19 vaccine is recommended in this population [26].

At the beginning of each influenza season, the Italian Ministry of Health defines the categories at higher risk of influenza complications, according to international recommendations of Public Health institutions [7], for which vaccination is to be offered actively and free of charge. For the 2021/2022 influenza season, the target categories included subjects aged ≥65 years [27].

In recent years, vaccination plans have begun to be individualized, paying particular attention to frail and older adults at higher risk for infectious diseases and distinguishing these individuals from older adults without a concomitant chronic condition; this is the case for COVID-19 vaccination. Despite this, knowledge of vaccine prophylaxis in higher-risk elderly is very limited; thus, there is an urgent need to better understand the complex interactions between age, comorbidities, and the immune system to develop more immunogenic vaccines and improved recommendations for older adults with comorbidities [6]. In fact, only a few clinical trials have investigated the efficacy of vaccines in the frail elderly [28,29,30,31,32]. This need became particularly pressing during the COVID-19 pandemic; indeed, it has been demonstrated that older adults are at higher risk of adverse outcomes and mortality because aging is associated with other conditions such as multimorbidity, frailty, and disability, as described above [33]. So, many authors have proposed that scientists, especially geriatricians, have much to contribute to the development, testing, and implementation of COVID-19 vaccines in this sub-group of populations, thereby pushing the field of vaccinology to embrace the complexity of frailty [34]. This consideration should be considered valid for all currently available preparations and for vaccines to be developed in the future.

The multifactorial approach should be implemented to achieve high immunization coverage in the elderly at highest infectious risk. The introduction of an intra-hospital protocol for the vaccination of chronic patients has been shown to strongly increase the VC (up to 10-fold) of these individuals [35]. For example, in the 2021/2022 influenza season, the Apulia region ordered the active offering of vaccination for hospitalized residents at greater risk of infectious disease [36]. Cooperation between the vaccinologist, physicians from other specialties, and general practitioners (GP) seems to be a determining factor in achieving better immunization rates in patients with higher infectious diseases. Actually, the lack of recommendation by the GP and the absence of a clear communication circuit between the GP and the branch specialist are the main obstacles in these patients’ access to immunization. Elderly patients with chronic disease(s) tend to identify the branch specialist as “their doctor” or “the most important doctor” even if they rarely discuss health issues that do not pertain to their area of specialization, such as vaccination prophylaxis and have a direct influence on the immunization decisions of their patients and social contacts [37]. In the Italian healthcare model (in which the patient is managed by both the GP and the specialist physician), it may not be clear who is responsible for recommending vaccination and what is the appropriate care setting to administer vaccines. Several studies have indicated the hospital as the ideal setting for actively offering vaccination in at-risk patients, in the context of inpatient or outpatient services [28].

Specific multidisciplinary teams could be built for preventive prophylaxis in nursing homes. One example would be the GIROT (Gruppo Intervento Rapido Ospedale Territorio) group in Tuscany, a multidisciplinary hospital-at-nursing-home team that aims to provide on-site intermediate care assistance to nursing home residents affected by COVID-19 [38]. This project, which has shown good results, could be extended to other vaccine-preventable infectious diseases, even outside the pandemic context. Moreover, the anti-COVID-19 vaccination campaign has allowed for the experimentation of on-site immunization of nursing home residents and long-term facilities [39], which could be repeated for other vaccines recommended for those patients.

Furthermore, vaccination hesitancy among older adults may be a determining factor in the success (or otherwise) of immunization campaigns. As a matter of fact, in 2019 the World Health Organization (WHO) listed vaccine hesitancy as a major threat to health that year [40]. Hence, healthcare professionals must be empowered to play their role in vaccination campaigns and their management; therefore, it is also necessary to work on the hesitancy of healthcare providers, considering that a hesitant provider is less likely to recommend the vaccine to his or her patients [41]. Social determinants also need to be considered; a 2017 systematic review [42] found that living alone, living in deprived areas, lower income, and lower education are associated with worse vaccination adherence in older adults. Thus, public health strategies must consider these elements as well.

In conclusion, scientific evidence and guidelines on the management of elderly patients at increased infectious risk are constantly evolving, although there is an almost complete absence of clinical trials or observational studies dedicated to this population category in the scientific literature. Therefore, scientific research needs to focus on the elderly at risk, so that recommendations can be tailored to ensure the best levels of efficacy and safety. On the other hand, the current recommendations in force need to be applied systemically; to do so, the role of health professionals in scientifically updating and recommending the right vaccine prophylaxis based on patient characteristics is fundamental, as is that of government institutions, which should implement policies focused on the population under study. The offer of free vaccine prophylaxis in the hospital with a close connection between the branch specialist and the vaccinologist would allow for concentrating on a single “hospital vaccine clinic” with various diagnostic and therapeutic paths that provide for the vaccination of subjects at risk for pathologies or conditions. In doing so, it is necessary to manage vaccine hesitation in elderly patients and take into account the social determinants that influence vaccine adhesion.

## Abbreviations

HCW	Healthcare worker
CDC	Centers for Disease Control and Prevention
WHO	World Health Organization
GP	General Practitioner
